# Acute and additive toxicity of ten photosystem-II herbicides to seagrass

**DOI:** 10.1038/srep17443

**Published:** 2015-11-30

**Authors:** Adam D. Wilkinson, Catherine J. Collier, Florita Flores, Andrew P. Negri

**Affiliations:** 1 College of Marine and Environmental Sciences, James Cook University, Townsville, Queensland, 4811, Australia; 2 Australian Institute of Marine Science, Townsville, Queensland, 4810, Australia; 3 Centre for Tropical Water & Aquatic Ecosystem Research (TropWATER), James Cook University, Cairns, Queensland, 4870, Australia

## Abstract

Photosystem II herbicides are transported to inshore marine waters, including those of the Great Barrier Reef, and are usually detected in complex mixtures. These herbicides inhibit photosynthesis, which can deplete energy reserves and reduce growth in seagrass, but the toxicity of some of these herbicides to seagrass is unknown and combined effects of multiple herbicides on seagrass has not been tested. Here we assessed the acute phytotoxicity of 10 PSII herbicides to the seagrass *Halophila ovalis* over 24 and/or 48 h. Individual herbicides exhibited a broad range of toxicities with inhibition of photosynthetic activity (*∆F/F*_*m*_′) by 50% at concentrations ranging from 3.5 μg l^−1^ (ametryn) to 132 μg l^−1^ (fluometuron). We assessed potential additivity using the Concentration Addition model of joint action for binary mixtures of diuron and atrazine as well as complex mixtures of all 10 herbicides. The effects of both mixture types were largely additive, validating the application of additive effects models for calculating the risk posed by multiple PSII herbicides to seagrasses. This study extends seagrass ecotoxicological data to ametryn, metribuzin, bromacil, prometryn and fluometuron and demonstrates that low concentrations of PSII herbicide mixtures have the potential to impact ecologically relevant endpoints in seagrass, including *∆F/F*_*m*_′.

Seagrass is found in coastal habitats globally, including all marine bioregions of Australia, and its distribution and form depends strongly on local environmental and anthropogenic conditions^1^. The estimated total area of seagrass meadows within the Great Barrier Reef (GBR) World Heritage Area is greater than 40,000 km^2^, and exceeds that of coral reef^2^,^3^. This together with their profound ecological importance highlights the significance of monitoring and protecting seagrass habitats^1^. One of the most widespread and common species throughout tropical and subtropical regions of Australia is *Halophila ovalis*^4^, with this species inhabiting the shallow zones to depths as great as 50 m^5^. *H. ovalis* is considered a colonising species^1^,^6^ with a high turnover in above-ground material^7^ and rapid re-growth when environmental conditions are again favourable^1^. Species such as *H. ovalis* may be considered a sentinel species as its sensitivity to environmental disturbances may provide insights or early warning of environmental stress (both seasonal and anthropogenic)^8^.

Photosystem II (PSII) herbicides are applied extensively to crops and the high mobility and persistence^9^ of these herbicides can result in elevated concentrations in the marine environment. For example, an estimated 30,000 kg per annum of PSII herbicides are transported through waterways into nearshore waters of the World Heritage listed GBR each year^10^. Herbicide concentrations are highest nearshore and in the vicinity of seagrass meadows^11^, which are sensitive to PSII herbicides^12^,^13^,^14^. These coastal seagrasses are also at risk from elevated turbidity from catchment and urban runoff, as well as port development^15^. Agricultural runoff from GBR catchments results in mixtures of PSII herbicides being detected at concentrations exceeding 0.9 μg l^−1^, the current 99% species protection guideline for the GBR^16^,^17^,^18^. These herbicides block electron transport in PSII of plants by binding to the D1 protein in the thylakoid membrane and displacing plastoquinone, which in turn inhibits the synthesis of ATP and NADPH^19^. The most sensitive indicator of PSII effects on marine organisms is the inhibition in effective quantum yield of PSII (Δ*F/F*_*m*_’) which can be measured using the non-invasive technique of pulse amplitude modulation (PAM) fluorometry (see Methods section)^20^. A reduction in Δ*F/F*_*m*_’ by PSII herbicides is directly linked to reduced photochemical efficiency^21^, and subsequently in seagrass to starvation and declines in growth and community fitness^13^,^20^,^22^. The ecological relevance of Δ*F/F*_*m*_’ inhibition as an endpoint in marine PSII herbicide toxicity studies is further demonstrated by its strong correlation with growth inhibition in microalgae^23^,^24^ and relationship with reduced reproductive output in corals^25^,^26^.

Diuron is one of the most potent PSII herbicides detected in marine and estuarine waters, inhibiting Δ*F/F*_*m*_’ by 50% (IC_50_) at between 2.1 and 3.5 μg l^−1^ in marine microalgae^23^, seagrass^8^,^12^,^13^,^27^ and corals^28^,^29^. The other most commonly detected PSII herbicide atrazine is on average ~8-fold less potent than diuron towards tropical seagrasses, corals, microalgae, foraminifera and crustose coralline algae^12^. The range of potencies among other PSII herbicides is wider still, with tebuthiuron, being approximately 14-fold less toxic than diuron to a similar range of non-target marine species^12^. The differences in potency among the PSII inhibitors is likely due to the diverse stearic and lipophilic properties of the herbicides^30^ along with other differences in structural interactions between the herbicides and the binding site in the D1 protein^19^. Changes in the usage patterns of PSII herbicides have led to an increasing diversity of herbicides being detected in the catchments and lagoon of the GBR^31^. While there may be sufficient ecotoxicological data to derive guidelines for some PSII herbicides such as, atrazine, ametryn, diuron, hexazinone and tebuthiuron^18^, little is known of the relative toxicity of other PSII herbicides, such as bromacil, prometryn, metribuzin and fluometuron, which have also been detected in the GBR or its catchments^32^,^33^,^34^,^35^,^36^. Understanding the relative toxicities of these alternative PSII herbicides to tropical marine species, such as seagrass, is important for the sustainability and management of agricultural practices adjacent to the GBR catchment area and other locations where PSII herbicides are detected^27^,^37^,^38^,^39^.

PSII herbicides are generally detected in complex mixtures with other PSII and/or non-PSII herbicides^40^,^41^,^42^. Although the PSII inhibitors are represented by a range of chemical classes (e.g. phenylurea, s-triazine and uracil), all have the same mode of action and their combined effects are considered additive for a variety of freshwater^24^,^43^,^44^,^45^ and estuarine microalgae^23^. The Concentration Addition (CA) model of joint action is valid for multiple PSII herbicides as this combines the concentration and potency of each component to calculate the expected total toxicity of a mixture^46^,^47^. A common approach to test the applicability of CA is to apply Toxic Unit (TU) values to the herbicide concentrations that induce the equivalent toxicity. For example, the concentration which inhibits Δ*F/F*_*m*_’ by 50% (IC_50_) = 1 TU and is different for each herbicide^23^,^45^. Concentration-response curves of herbicide mixtures containing a range of TU values can then be used to validate CA for combinations of herbicides in a mixture (see Methods section).

Since the values of the World Heritage listed GBR are based on the fitness and survival of foundation, habitat-forming species such as seagrass, ecotoxicological data for these species should be included in future risk assessments and for the derivation and assessment of future water quality guidelines^48^. Here we assess the acute toxicity of 10 PSII herbicides (Table 1) individually and in mixtures to the tropical seagrass species, *Halophila ovalis*. The acute effects of individual herbicides on photosynthetic performance (*∆F/F*_*m*_’) of isolated leaves using a miniature bioassay was assessed in concentration-response experiments over a 24 and/or 48 h exposure period(s) in a static system^8^. The miniature bioassay methodology was also applied to examine the toxicity of PSII herbicides in binary and complex mixtures. Data from this study will broaden the relevant ecotoxicity data to include a range of alternative and emerging PSII herbicides and validate the additive toxicity of PSII herbicide mixtures on seagrass for application in monitoring programs and guideline development.

## Results

### Potencies of individual herbicides

**Figure 1:**
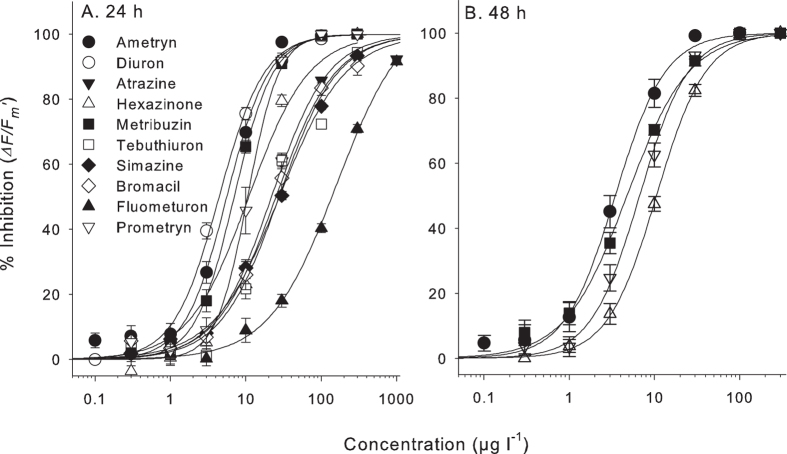
Concentration-response curves for individual herbicides. Concentration response curves for inhibition of *∆F/F*_*m*_’ measured at (**A**) 24 h and (**B**) 48 h for 10 individual herbicides, relative to each solvent control. The four herbicides in (**B**) did not reach maximum inhibition of *∆F/F*_*m*_’ within 24 h. Bars represent SE ± n = 9.

All herbicides tested inhibited *∆F/F*_*m*_’ in *H. ovalis* enabling classical concentration-response relationships to be fitted (Fig. 1) with high levels of confidence (r^2^ values = 0.98–0.99). The herbicide concentrations that inhibited *∆F/F*_*m*_’ by 10% (IC_10_) and 50% (IC_50_) are listed in Table 2. After 24 h, diuron was the most potent of the herbicides, exhibiting the lowest IC_50_ of 4.3 μg l^−1^ (Table 2). Fluometruon with an IC_50_ of 132 μg l^−1^ was the least potent of the herbicides tested in the 24 h assays. Maximum inhibition of *∆F/F*_*m*_’ was reached before 24 h for all herbicides^8^ apart from ametryn, metribuzin, prometryn and hexazinone which reached maximum inhibition by 48 h. This additional 24 h of exposure resulted in lower IC_50_ values, reducing them by a further 63% to 69% (Fig. 1B and Table 2). The potencies for each of the herbicides can be evaluated using the Relative Potencies (ReP) compared to the reference herbicide diuron (IC_50_ diuron/IC_50_ herbicide) (Table 2). ReP values > 1 indicate potencies proportionally greater than diuron and ReP values < 1 indicate potencies less than diuron.

### Mixture toxicity

**Figure 2:**
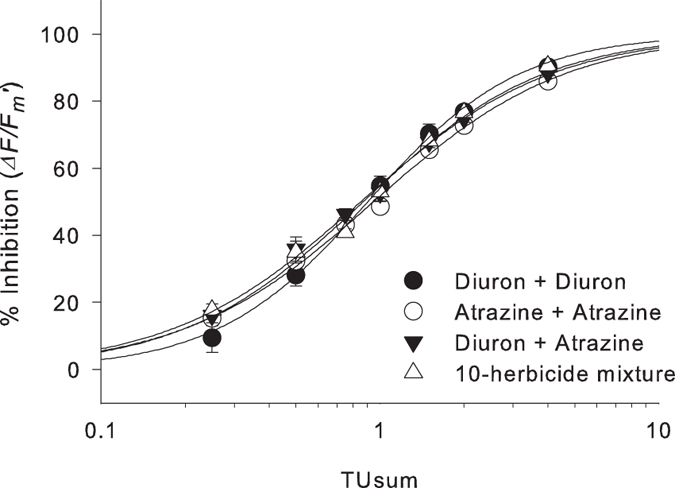
Concentration-response curves for inhibition of *∆F/F*_*m*_’ by herbicide mixtures. Inhibition was measured at 24 h for binary and complex (10) herbicide mixtures, relative to each solvent control. Bars represent SE ± n = 9.

The response of *H. ovalis* to the four mixtures tested (binary and complex) were also plotted as concentration-response curves (Fig. 2). The four curves largely overlapped across the range of Toxic Units (TUs) indicating little difference in the response of *∆F/F*_*m*_’ between the different mixtures and this was confirmed by the calculated IC_50_ which ranged between 0.85 TU – 0.95 TU (3). For additivity using the Concentration Addition (CA) method, the IC_50_ of each of the mixtures would be expected to be close to 1 TU, which was determined by the individual concentration-responses (Table 2). The reference mixtures of [diuron + diuron] and [atrazine + atrazine] exhibited IC_50_s of 0.90 TU – 0.95 TU indicating slightly more sensitive responses to both herbicides than was observed during the individual herbicide assays. F-test analysis indicated a significant difference within the 4-way mixture comparison (F_3,24_ = 3.21, p < 0.05). The post-hoc analysis indicated that the IC_50_ of [diuron + atrazine] was slightly (11%) but significantly lower (i.e. more potent) than the IC_50_ of [atrazine + atrazine] (Table 3). This indicates a possible synergistic interaction; however, there was no significant difference between the IC_50_ of the [diuron + atrazine] mixture and the other mixtures (Table 3).

## Discussion

Phytotoxicity in non-target plants, such as seagrass, has been documented previously for the PSII herbicide diuron in several studies^8^,^12^,^13^,^49^ and its effects in chronic exposures lead to both declines in stored energy in the root-rhizome complex and whole-plant effects, including reduced growth and survival^13^. Here we extend the toxic threshold (IC_10_) and comparative toxicity data (IC_50_) for inhibition of photosynthesis (*∆F/F*_*m*_’) in *H. ovalis* to a further nine PSII herbicides, and this matched dataset includes the first ecotoxicological information for ametryn, metribuzin, bromacil, prometryn and fluometuron for any seagrass species. Confirmation of additive toxicity of binary and complex PSII herbicide mixtures to *H. ovalis* further validates the importance of additive ecotoxicological effects (when the mode of action is the same) for application in field monitoring, water quality guideline development and in ecological risk assessments.

### Herbicide potencies

The PSII herbicides demonstrated a wide range of potencies with diuron being most toxic (IC_50_ = 4.3 μg l^−1^) and all other herbicides exhibiting IC_50_s < 30 μg l^−1^ except fluometuron which was four-fold less toxic than all other herbicides after 24 h (Table 2). All of these herbicides bind to the same site in the D1 protein^19^ and differences in potency are likely due to the diverse stearic, and lipophilic properties of the herbicides, where herbicides “fit” and form different covalent attachments with the protein^30^. We previously demonstrated even uptake and binding of diuron through the leaf surface of *H. ovalis* using Imaging-PAM fluorometry and no flooding of the vascular system via the cut stems of isolated *H. ovalis* leaves^8^. Herbicides with different structures and hydrophobicity are likely to be transported through the leaf and to and from the binding site at various rates, potentially contributing to less rapid impacts of ametryn, metribuzin, prometryn and hexazinone (Table 2). PSII herbicides must cross the hydrophobic semi-permeable cell membrane of the cell in order to successfully inhibit photosynthetic function, and absorption may be more difficult for less lipophilic herbicides^19^ such as hexazinone. These slow acting herbicides here are all related s-triazines or triazinones, but the group exhibits a wide range of water solubilities and lipophilicities (Table 1).

This study provides the first seagrass phytotoxicity data for fluometuron, ametryn, metribuzin, prometryn and bromacil, and builds on limited toxicological data for atrazine, hexazinone, simazine and tebuthiuron to tropical species (Table 4). *H. ovalis* was generally more sensitive to many of these PSII herbicides when compared to other species groups (Table 4), though with some exceptions. Atrazine for example inhibited *∆F/F*_*m*_’ at similar concentrations in *H. ovalis* as for other seagrass species in 3 day exposures of potted plants^12^, but at lower concentrations (i.e. greater sensitivity) than green algae^45^,^50^ or coral^28^,^51^ (Table 4). *H. ovalis* was also more sensitive to simazine than green algae^45^, coral^28^ and diatoms^52^. Despite differences in sensitivity of *∆F/F*_*m*_’ inhibition between species to the same herbicide, these differences were usually within an order of magnitude due to the well conserved binding site on the D1 protein in PSII^53^. Differences in experimental conditions including temperature^29^, light levels^8^ and exposure time^13^ between studies are also likely to affect apparent toxicity, highlighting the need for strictly controlled and repeatable experimental procedures in phytotoxicity studies.

### Application to water quality guidelines

Ecotoxicity threshold values (ETVs) developed specifically for the GBR are intended to protect 99% of species in the World Heritage Area; however, these were developed from limited toxicity data^18^ (Table 5). Inhibition of *∆F/F*_*m*_’ is directly and quantitatively linked to inhibition of photochemical efficiency^54^ and this in turn leads to reduced energy status and/or growth and mortality in seagrass following chronic PSII exposures^13^,^20^,^22^. Inhibition of (*∆F/F*_*m*_’) is also well correlated with reduced growth in microalgae^23^,^24^ and energetics and reproduction in corals^25^,^26^ and can therefore be considered ecologically relevant as a basis from which guidelines can be developed or assessed. Five of the herbicides registered for use in catchments of the GBR and tested here have no current guidelines; therefore, the matched IC_10_ and IC_50_ data (Table 2) provides valuable toxicity data as a contribution to risk assessments, interpretation of water quality monitoring and derivation of future guidelines. For some of the herbicides, greater than 10% inhibition of seagrass photosynthesis occurred at concentrations lower than current and proposed ETVs (Table 5).

### Mixture toxicity

The overlapping concentration-response curves of all PSII herbicide mixtures and similarity between IC_50_s (TU_atr+diu_ of 0.85 was only 6% and 11% lower than either TU_diu+diu_ or TU_atr+atr_) indicates additivity of herbicide effects on PSII activity in *H. ovalis* (Table 3). The small but significant difference between IC_50_ values for [atrazine + diuron] and [atrazine + atrazine] indicated a potentially weak synergistic effect, but no differences between IC_50_ for the 10-herbicide mixture (TU_mix_) and either of the controls was evident, supporting overall additivity. These results build upon previous research demonstrating the validity of additive effects of PSII herbicide mixtures on photosynthesis with estuarine microalgae in the laboratory^23^ and in microcosms^55^, and on cell division in the freshwater green algae *Scenedesmus acuolatus* for multiple complex mixtures of up to 18 *s*-triazines^45^. While Concentration Addition (CA) model of joint action is an appropriate approach for calculating total toxicity in mixtures of toxins with the same mode of action (such as PSII herbicides), alternative approaches should be applied to mixtures containing PSII herbicides and pesticides with other modes of action^56^. Contributions towards total toxicity by multiple PSII herbicides, each acting simultaneously at concentrations below individual guidelines can result in ecologically significant effects on aquatic organisms^45^ and water quality guidelines based on single herbicides, even widespread and potent herbicides like diuron, could underestimate the ecological threat posed by herbicide mixtures. Concentration Addition has already been applied to compare the actual and expected additive phytotoxicity of field samples containing more than one PSII herbicide^57^,^58^,^59^. CA has also been applied to calculate total toxicity for complex mixtures of PSII herbicides in the field towards guideline reporting and risk assessments^16^,^17^,^33^ and the current study validates this approach for PSII herbicides and ecologically important seagrass species. Matched ecotoxicity datasets like this one for multiple PSII herbicides are valuable, not only for comparing toxicities of individual herbicides but are critical for direct application in evaluating the total toxicity and risks posed by mixtures that are commonly observed in the environment such as the GBR and its catchments^16^,^17^,^60^.

## Methods

### Seagrass collection and preparation

*H. ovalis* plants were collected at low tide in intertidal meadows of Cockle Bay, Magnetic Island (19°10.88′S, 146°50.63′E) under Permit MTB41 (Department of Employment, Economic Development and Innovation). Small plugs of seagrass with 5–10 cm (depth) of associated sediment was extracted and placed in plastic plant pots lined with plastic bags. The bag was pulled up over the seagrass with a small amount of water retained and secured for transport. Plants were taken to the Australian Institute of Marine Science (AIMS), Townsville, Queensland and placed into 60 l aquaria within 4 h from collection under moderate illumination (270–300 μmol photons m^−2^s^−1^). Water temperature conditions (25–28 °C) and salinity (34–36 ppt) were maintained throughout the acclimation phase.

### Herbicides

Photosystem II inhibiting herbicides from four chemical classes (Table 1) were tested individually and in combination for their toxicity to seagrass. This selection of herbicides was based on application rates as well as contamination data in Queensland catchments adjacent to the GBR^14^,^16^,^33^,^41^,^42^. The herbicide diuron was included as a reference toxicant^24^. All herbicides were purchased in the purest available analytical form (>95%) from Sigma Aldrich. Individual herbicide solutions were prepared in 0.2 μm filtered seawater using ethanol as a carrier (<0.03% v/v). Nominal concentrations are reported as the herbicides are non-volatile, water solubility >30 mg l^−1^ and octanol-water coefficient (log K_ow_) <4 making loss to adsorption on test vessels unlikely^61^,^62^. The measured seawater pH, salinity and oxygen concentrations in tests were 8.1, 34–36 psu and 7.0–8.5 mg l^−1^ respectively.

### Miniature seagrass leaf assay

Assays were conducted in 12-well plates (Nunclon, Thermo Scientific), each containing 5 ml herbicide solution. Herbicide concentrations were randomized across all plates to minimise well cluster and potential plate effects^8^. Experimental light intensity was 100 ± 7 μE (14:10 h light:dark cycle) and temperature maintained at 26 ± 2 °C for all assays. Fluorescence measurements were made with a MAXI Imaging-PAM (I-PAM) (Walz, Germany).

Two fluorescence parameters were used to assess impacts of PSII herbicides on the seagrass leaves^8^,^20^. The effective quantum yield in an illuminated plant (∆*F*/*F*_*m*_’) provides an estimate of the efficiency of photochemical energy conversion within PSII under a given light intensity^54^. The maximum quantum yield (*F*_v_/*F*_m_) is equivalent to the proportion of light used for photosynthesis by chlorophyll when all reaction centres are open^54^ and reductions in *F*_v_/*F*_m_ indicate inactivation and/or photo-oxidative damage to PSII (chronic photoinhibition)^63^.

To quantify *∆F/F*_*m*_’, actinic light (100 ± 3 μE) was applied within the I-PAM chamber for five minutes prior to the activation of the saturating pulse. Minimum fluorescence (*F* with illuminated samples) was determined by applying a weak modulated blue measuring light (ML setting of 5; 650 nm, 0.15 μmol photons m^−2^s^−1^). Light adapted maximum fluorescence (*F*_*m*_’) was determined using a short pulse (800 ms) of saturating actinic light (>3000 μmol photons m^−2^s^−1^) and the effective quantum yield of PSII calculated from *∆F/F*_*m*_’ = (*F*_*m*_’ – *F*)/*F*_*m*_’. To quantify *F*_*v*_*/F*_*m*_, leaves were dark adapted for 30 min and *F*_*0*_ and *F*_*m*_ measured in the same fashion as *F* and *F*_*m*_’ to derive maximum quantum yields *F*_*v*_*/F*_*m*_ = (*F*_*m*_ – *F*_*0*_)/*F*_*m*_. *F*_v_*/F*_m_. Inhibition of quantum yields (% inhibition relative to solvent control) was calculated from treatment data as Inhibition (%) = [(Y_control_−Y_sample_)/Y_control_] × 100, where Y is Δ*F/F*_*m*_’ or *F*_*v*_*/F*_*m*_.

### Screening

A screening process was performed immediately prior to running the assays to ensure the leaves were in optimal condition for the experiment^8^. Second and third leaf pairs from the terminal, apical end of the rhizome were transferred to wells containing uncontaminated seawater. Leaves were dark adapted for 30 min and *F*_*v*_*/F*_*m*_ was measured. Only leaves exhibiting *F*_*v*_*/F*_*m*_ greater than 0.65 (indicating intact and efficient photosystem II apparatus) were used in the subsequent herbicide assays^8^. Average leaf length was 10.0 mm ± 2.5 and width was 4.8 mm ± 1.2.

### Experimental duration and leaf health

*F*_*v*_*/F*_*m*_ was measured at 0, 24 and 48 h to assess whether PSII remained intact and active^8^. The maximum fluorescence yield (*F*_*v*_*/F*_*m*_) in uncontaminated solvent controls reduced by less than 8.5% over 24 and 48 h durations in all experiments, confirming that PSII remained intact and functional over the assay duration (one-way ANOVA p =  < 0.05). Maximum inhibition of *∆F/F*_*m*_’ in *H. ovalis* leaves by diuron is observed in less than 24 h^8^. Here, range finding exposures were performed for all other herbicides to determine whether maximum inhibition of *∆F/F*_*m*_’ would be achieved following 24 or 48 h exposures. Leaves were exposed to high concentrations of each herbicide and the exposure duration to reach 95% steady state inhibition was recorded. Maximum inhibition was reached between 12 and 24 h exposure for all herbicides except hexazinone, metribuzin, prometryn and ametryn, which were reached within 48 h.

### Concentration-response curves

Concentration-response curves were plotted by fitting four parameter logistic curves to the *∆F/F*_*m*_’ inhibition data from nine replicate leaves for each concentration (SigmaPlot 11.0 and Graph Pad Prism V 6.0). Herbicide concentrations inhibiting *∆F/F*_*m*_’ by 10 and 50% (IC_10_ and IC_50_) were determined from each curve by applying standard curve analysis. The probability that midpoints (IC_50_) generated by the logistic curves were statistically different was tested by applying the F test in Graph Pad Prism V 6.0. IC_50_s were considered different when p < 0.05 and post-hoc results are presented for each comparison in the relevant results sections.

### Mixture toxicity

Concentration addition (CA) was tested for (i) a binary mixture of [diuron and atrazine] (each 50% v:v) and (ii) a mixture of all [10 herbicides] (each 10% v-v). The Toxic Units (TU) concentration for each component was based on its IC_50_ (=1 TU) at 24 h calculated from the individual assays (Table 3). The bioassay was prepared and conducted in an identical way to solitary herbicide assays (see above). TU_sum_ was calculated from corresponding TU values within the mixture (see Eq 1).




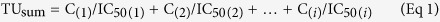




C_(i)_ refers to the concentration of the *ith* herbicide in the mixture. Expected mixture toxicity is derived from TU_sum_ data and compared directly to experimental data. If 50% inhibition *∆F/F*_*m*_’ of the mixture was reached at 1 TU (IC_50_) the effect is additive. If 50% inhibition is obtained at a < 1 TU_sum_ the effect is considered synergistic and if it is reached at > 1 TU_sum_ the mixture toxicity is classified as antagonistic^23^. A TU dilution series was applied to all herbicide mixtures (0, 0.25, 0.5, 0.75, 1, 1.5, 2 and 4 TU). The binary mixtures of (i) [diuron + atrazine] and (ii) [10 herbicides] were compared against duplicate reference mixtures of [diuron + diuron] (50% v/v mixture) and [atrazine + atrazine] (50% v/v mixture) to confirm additivity. Additivity was considered true when the observed mean IC_50_ TU_sum_ was close to unity and not significantly different to the average IC_50_ of the mixture control response. Differences between IC_50_s were tested using the F-test in GraphPad V6.0.

## Additional Information

**How to cite this article**: Wilkinson, A. D. *et al.* Acute and additive toxicity of ten photosystem-II herbicides to seagrass. *Sci. Rep.* **5**, 17443; doi: 10.1038/srep17443 (2015).

## Figures and Tables

**Table 1:** Properties of herbicides tested.

Herbicide	Chemical class	Log K_ow_	Water solubility (mg l^−1^)	CAS number
Diuron	phenylurea	2.6	37.4	330-54-1
Fluometuron	Phenylurea	2.4	110	2164-17-2
Tebuthiuron	Phenylurea	1.8	2,500	34014-18-1
Atrazine	s-triazine	2.5	29,800	1912-24-9
Ametryn	s-triazine	2.6	200	834-12-8
Metribuzin	s-triazine	1.6	1050	21087-64-9
Simazine	s-triazine	2.1	6.2	122-34-9
Prometryn	s-triazine	3.1	33	7287-19-6
Bromacil	uracil	1.9	807	317-40-9
Hexazinone	triazinone	1.2	33,000	51235-04-2

Water solubility calculated at > 20 °C. All data from^64^.

**Table 2:** Phytotoxicity inhibition endpoints for individual herbicides after 24 and 48 h exposures.

Herbicide	EC_10_	95% CI	EC_50_ = (1 TU)	95% CI	R^2^	ReP
*24* *h exposures*						
Diuron	1.2	1.0–1.5	4.3	3.9–4.7	0.99	1.0
Fluometuron	17	13–22	132	116 –150	0.99	0.033
Tebuthiuron	3.9	2.9–5.2	28	24–31	0.98	0.16
Atrazine	3.4	2.8–4.0	22	20–24	0.99	0.19
Ametryn	1.4	1.1–1.8	5.6	4.9–6.3	0.99	0.77
Metribuzin	1.9	1.6–2.3	7.0	6.5–7.5	0.99	0.61
Simazine	3.0	2.5–3.6	28	26–30	0.99	0.15
Prometryn	3.7	2.8–4.9	11	9.4–12	0.99	0.41
Bromacil	3.4	2.6–4.6	25	22–29	0.99	0.17
Hexazinone	6.4	5.6–7.3	17	16–18	0.99	0.26
*48* *h exposures*						
Ametryn	0.8	0.6–1.0	3.5	3.0–4.0	0.99	1.23
Metribuzin	0.8	0.6–1.0	4.8	4.3–5.3	0.99	0.90
Prometryn	1.6	1.3–2.0	6.7	6.0–7.5	0.99	0.64
Hexazinone	2.5	2.0–3.0	11	9.7–12	0.99	0.41

Inhibition of *∆F/F*_*m*_’ IC_10_ and IC_50_ data (μg l^−1^) with 95% confidence intervals. Toxic units for the mixture experiments were assigned as 1 TU = IC_50_ at 24 h. Relative potencies (ReP), relative to diuron (IC_50_ diuron/IC_50_ herbicide).

**Table 3:** A comparison of additive toxicity of binary and complex mixtures.

Herbicide mixture	IC_10_ (TU_sum_)	95% CV	IC_50_ (TU_sum_)	95% CV
Diuron + diuron	0.23	0.20–0.27	0.90^a,b^	0.87–0.94
Atrazine + atrazine	0.17	0.14–0.20	0.95^a^	0.90–1.0
Diuron + atrazine	0.15	0.12–0.18	0.85^b^	0.81–0.90
10-herbicide-mix	0.17	0.14–0.20	0.87^a,b^	0.8–0.95

*∆F/*_*Fm*_’ IC_10_ and IC_50_ data (TU) of all four herbicide mixtures after 24 hr exposures. The proportions of each mixture are equal. For example the binary mixtures contain 50% v/v of each component while the 10-herbicide mix comprises 10% v/v of each herbicide. Different letters in superscript indicate significant differences in IC_50_ (p < 0.05). Note all IC_x_ values are listed as TU_sum_ values not concentrations.

**Table 4:** A summary of relevant toxicity data for the 10 PSII herbicides tested towards a range of species.

Herbicide	Duration	Test phylum	Common name	Indicator Endpoint	Response concentration	Reference
Diuron	24 h	Angiospermae	Seagrass	∆F/F_m’_ (IC_50_)	4.3 μg l^−1^	Present study
	24 h	Angiospermae	Seagrass	∆F/F_m’_ (IC_50_)	3.5 μg l^−1^	8
	72 h	Angiospermae	Seagrass	∆F/F_m’_/F_v_/F_m_	2.4–2.47 μg l^−1^	12
	5 day	Angiospermae	Seagrass	∆F/F_m’_ (LOEC)	0.1 μg l^−1^	65
	4 days	Angiospermae	Seagrass	∆F/F_m’_ (LOEC)	10 μg l^−1^	49
	77 days	Angiospermae	Seagrass	∆F/F_m’_ (IC_50_)	2.4–2.8 μg l^−^1	13
	34 h	Dinoflagellate	Coral	∆F/F_m’_ (IC_50_)	2.9–5.9 μg l^−1^	51
	2–3 mo	Dinoflagellate	Coral	∆F/F_m’_ (IC_50_)	1.2–5.0 μg l^−1^	66
	4 day	Heterokontophceae	Diatom	∆F/F_m’_ (IC_50_)	2.6–18 μg l^−1^	23
	4 day	Chlorophyceae	Green algae	∆F/F_m’_ (IC_50_)	2.1 μg l^−1^	23
Fluometuron	24 h	Angiospermae	Seagrass	∆F/F_m’_ (IC_50_)	132 μg l^−1^	Present study
	30 min + 48 h	Chlorophyceae	Green algae	Growth	2.5–10 ml l^−1^	67
Tebuthiuron	24 h	Angiospermae	Seagrass	∆F/F_m’_ (IC_50_)	28 μg l^−1^	Present study
	72 h	Angiospermae	Seagrass	∆F/F_m’_ (IC_50_)	29.1–29.7 μg l^−1^	12
	24 h	Dinoflagellate	Coral	∆F/F_m’_ (IC_50_)	175 μg l^−1^	68
	4 day	Heterokontophceae	Diatom	∆F/F_m’_ (IC_50_)	51–94 μg l^−1^	23
	4 day	Chlorophyceae	Green algae	∆F/F_m’_ (IC_50_)	12 μg l^−1^	23
Atrazine	24 h	Angiospermae	Seagrass	∆F/F_m’_ (IC_50_)	22 μg l^−1^	Present study
	72 h	Angiospermae	Seagrass	∆F/F_m’_ (IC_50_)	13.4–18.2 μg l^−1^	12
	96 h	Angiospermae	Seagrass	∆F/F_m’_ (LOEC)	10 μg l^−1^	49
	14 d	Angiospermae	Aquatic plants	∆F/F_m’_ (IC_50_)	22–132 μg l^−1^	50
	96 h	Chlorophyceae	Green algae	∆F/F_m’_ (IC_50_)	94–176 μg l^−1^	50
	24h	Chlorophyceae	Green algae	∆F/F_m’_ (IC_50_)	38.8 μg l^−1^	45
	2 h	Chlorophyceae	Green algae	∆F/F_m′_ (IC_50_)	103 μg l^−1^	52
	2 h	Heterokontophceae	Diatom	∆F/F_m’_ (IC_50_)	45 μg l^−1^	52
	24 h	Dinoflagellate	Coral	∆F/F_m’_ (IC_50_)	45 μg l^−1^	68
	34 h	Dinoflagellate	Coral	∆F/F_m’_ (IC_50_)	37 – 88.2 μg l^−1^	51
	4 day	Heterokontophceae	Diatom	∆F/F_m’_ (IC_50_)	34–77 μg l^−1^	23
	4 day	Chlorophyceae	Green algae	∆F/F_m’_ (IC_50_)	14 μg l^−1^	23
Ametryn	48 h	Angiospermae	Seagrass	∆F/F_m′_ (IC_50_)	3.6 μg l^−1^	Present study
	24 h	Chlorophyceae	Green algae	∆F/F_m’_ (IC_50_)	3.6 μg l^−1^	45
	24 h	Dinoflagellate	Coral	∆F/F_m’_ (IC_50_)	1.7 μg l^−1^	68
Metribuzin	48 h	Angiospermae	Seagrass	∆F/F_m′_ (IC_50_)	4.8 μg l^−1^	Present study
	14 d	Angiospermae	Aquatic plants	∆F/F_m’_ (IC_50_)	14–36 μg l^−1^	50
	h - days	Chlorophyceae	Green algae	∆F/F_m’_ (IC_50_)	12.3 – 39.7 μg l^−1^	69
	96 h	Chlorophyceae	Green algae	∆F/F_m’_ (IC_50_)	23–152 μg l^−1^	50
Simazine	24 h	Angiospermae	Seagrass	∆F/F_m’_ (IC_50_)	28 μg l^−1^	Present study
	96 h	Angiospermae	Seagrass	∆F/F_m’_ (LOEC)	10 μg l^−1^	49
	24 h	Chlorophyceae	Green algae	∆F/F_m’_ (IC_50_)	56.9 μg l^−1^	45
	24 h	Dinoflagellate	Coral	∆F/F_m’_ (IC_50_)	150 μg l^−1^	68
	2 h	Chlorophyceae	Green algae	∆F/F_m’_ (IC_50_)	76 μg l^−1^	52
	2 h	Heterokontophceae	Diatom	∆F/F_m’_ (IC_50_)	400 μg l^−1^	52
Prometyrn	48 h	Angiospermae	Seagrass	∆F/F_m’_ (IC_50_)	6.7 μg l^−1^	Present study
	24 h	Chlorophyceae	Green algae	∆F/F_m’_/F_v_/F_m_	13.2 μg l^−1^	45
Bromacil	24 h	Angiospermae	Seagrass	∆F/F_m′_ (IC_50_)	25 μg l^−1^	Present study
	2 h	Phaeophyceae	Macroalgae	∆F/F_m’_ (IC_50_)	8.23 μg l^−1^	70
Hexazinone	48 h	Angiospermae	Seagrass	∆F/F_m′_ (IC_50_)	11 μg l^−1^	Present study
	72 h	Angiospermae	Seagrass	∆F/F_m’_/F_v_/F_m_	4.4–6.9 μg l^−1^	12
	24 h	Dinoflagellate	Coral	∆F/F_m′_/F_v_/F_m_	8.8 μg l^−1^	68
	2 h	Chlorophyceae	Green algae	∆F/F_m’_/F_v_/F_m_	21 μg l^−1^	52
	2 h	Heterokontophceae	Diatom	∆F/F_m’_ (IC_50_)	22 μg l^−1^	52
	4 day	Heterokontophceae	Diatom	∆F/F_m′_ (IC_50_)	5.7–6.9 μg l^−1^	23
	4 day	Chlorophyceae	Green algae	∆F/F_m’_ (IC_50_)	2.4 μg l^−1^	23

Lowest observed effect concentration (LOEC).

**Table 5:** Comparison between IC_10_ and IC_50_ values and relevant ecological guidelines.

Herbicide	IC_10_	IC_50_	ANZECC^71^ ETV 99%,95%,90%	Proposed ANZECC^72^ ETV 99%,95%,90%	GBRMPA^18^ ETV 99%,95%,90%
Diuron	1.2	4.3	**1.8, 1.8, 1.8**	0.08, 0.3, 0.4	0.9, **1.6, 2.3**
Fluometuron	17	132	NA	NA	NA
Tebuthiuron	3.9	28	0.02, 2.2, **20**	**4.3, 8.8, 12.0**	0.02, 2, 20
Atrazine	3.4	22	0.7, **13, 45**	2.8, **3.8, 4.6**	0.6, 1.4, 2.5
Ametryn*	0.8	3.5	NA	0.02, 0.1, 0.3	0.5, **1.0, 1.6**
Metribuzin*	0.8	4.8	NA	NA	NA
Simazine	3.0	28	NA	NA	0.2, 3.2, 11
Prometryn*	1.6	6.7	NA	NA	NA
Bromacil	3.4	25	NA	NA	NA
Hexazinone*	2.5	11	**75, 75, 75**	0.9, 1.2, 1.5	1.2, 1.2, 1.2

All concentrations in μg l^−1^. Ecotoxicity threshold values (ETVs μg l^−1^) formulated to protect 99%, 95%, 90% of phototropic species. Current ANZECC guidelines are freshwater. NA not available. ETVs in bold are not protective of PSII activity in *H. ovalis* at the IC_10_ threshold. *indicates 48 h IC_50_ values.

## References

[b1] Kilminster K. *et al.* Unravelling complexity in seagrass systems for management: Australia as a microcosm. Sci. Total Environ. 15, 97–109 (2015). 25917445 10.1016/j.scitotenv.2015.04.061

[b2] Coles R., McKenzie L., De’ath G., Roelofs A. & Lee Long W. Spatial distribution of deepwater seagrass in the inter-reef lagoon of the Great Barrier Reef World Heritage Area. Mar. Ecol. Prog. Ser. 392, 57–68 (2009).

[b3] McKenzie L., Yoshida R., Grech A. & Coles R. *Queensland Seagrasses. Status 2010-Torres Strait and East Coast*, Fisheries Queensland (DEEDI), Cairns. 6 pp. (2010).

[b4] Waycott M., McMahon K. M., Mellors J. E., Calladine A. & Kleine D. A guide to tropical seagrasses of the Indo-West Pacific. 72 pp (James Cook University, 2004).

[b5] Lee Long W. J., Coles R. G. & McKenzie L. J. *Deepwater seagrasses in Northeastern Australia-How deep, how meaningful.* Seagrass Biology: Proceedings of an International Workshop, Rottnest Island, Western Australia. p 25–29 (January, 1996).

[b6] Rasheed M. A. Recovery and succession in a multi-species tropical seagrass meadow following experimental disturbance: the role of sexual and asexual reproduction. J. Exp. Mar. Biol. Ecol. 310, 13–45 (2004).

[b7] Duarte C. M. & Chiscano C. L. Seagrass biomass and production: a reassessment. Aquat. Bot. 65, 159–174 (1999).

[b8] Wilkinson A. D. *et al.* A miniature bioassay for testing the acute phytotoxicity of photosystem II herbicides on seagrass. PLoS ONE 10, e0117541 (2015). 25674791 10.1371/journal.pone.0117541 PMC4326278

[b9] Mercurio P., Mueller J. G. E., Flores F. & Negri A. Herbicide persistence in seawater simulation experiments. PLoS ONE 10, e0136391 (2015). 26313296 10.1371/journal.pone.0136391 PMC4552293

[b10] Kroon F. J. *et al.* River loads of suspended solids, nitrogen, phosphorus and herbicides delivered to the Great Barrier Reef lagoon. Mar. Pollut. Bull. 65, 167–181 (2012). 22154273 10.1016/j.marpolbul.2011.10.018

[b11] Lewis S. *et al.* Assessing the risk of additive pesticide exposure in Great Barrier Reef ecosystems in Assessment of the relative risk of water quality to ecosystems of the Great Barrier Reef: Supporting Studies (eds Waterhouse J. *et al.* ) Ch 4, 128 p. (Department of the Environment and Heritage Protection, Queensland Government, 2013).

[b12] Flores F., Collier C. J., Mercurio P. & Negri A. P. Phytotoxicity of four photosystem II herbicides to tropical seagrasses. PLoS ONE 8, e75798 (2013). 24098726 10.1371/journal.pone.0075798 PMC3786934

[b13] Negri A. P., Flores F., Mercurio P., Mueller J. F. & Collier C. J. Lethal and sub-lethal chronic effects of the herbicide diuron on seagrass. Aquat. Toxicol. 165, 73–83 (2015). 26026671 10.1016/j.aquatox.2015.05.007

[b14] Haynes D., Müller J. & Carter S. Pesticide and herbicide residues in sediments and seagrasses from the Great Barrier Reef World Heritage Area and Queensland coast. Mar. Pollut. Bull. 41, 279–287 (2000).

[b15] Grech A., Coles R. & Marsh H. A broad-scale assessment of the risk to coastal seagrasses from cumulative threats. Mar. Policy 35, 560–567 (2011).

[b16] Smith R. *et al.* Large-scale pesticide monitoring across Great Barrier Reef catchments – Paddock to Reef Integrated Monitoring, Modelling and Reporting Program. Mar. Pollut. Bull. 65, 117–127 (2012). 21920563 10.1016/j.marpolbul.2011.08.010

[b17] Lewis S. E. *et al.* Assessing the additive risks of PSII herbicide exposure to the Great Barrier Reef. Mar. Pollut. Bull. 65, 280–291 (2012). 22172236 10.1016/j.marpolbul.2011.11.009

[b18] GBRMPA. Water quality guidelines for the Great Barrier Reef Marine Park (Revised). Great Barrier Reef Marine Park Authority, Townsville. (2010) Available: http://www.gbrmpa.gov.au/corp_site/key_issues/water_quality/water_quality_guidelines. Accessed: 22 October 2015.

[b19] Oettmeier W. Herbicides of photosystem II In The Photosystems: Structure, Function and Molecular Biology (ed Barber J. ) 349–408 (Elsevier, 1992).

[b20] Ralph P. J., Smith R. A., Macinnis-Ng C. M. O. & Seery C. R. Use of fluorescence-based ecotoxicological bioassays in monitoring toxicants and pollution in aquatic systems: Review. Toxicol. Environ. Chem. 89, 589–607 (2007).

[b21] Schreiber U., Bilger W. & Neubauer C. Chlorophyll fluorescence as a non-intrusive indicator for rapid assessment of *in vivo* photosynthesis in *Ecophysiology of Photosynthesis* (eds Schulze E. D. & Caldwell M. M. ) 49–70 (Springer-Verlag, 1994).

[b22] Wahedally S. F., Mamboya F. A., Lyimo T. J., Bhikajee M. & Björk M. Short-term effects of three herbicides on the maximum quantum yield and electron transport rate of tropical seagrass *Thalassodendron ciliatum*. Tanzania J. Agric. Sci. 3, 458–466 (2012).

[b23] Magnusson M., Heimann K., Quayle P. & Negri A. P. Additive toxicity of herbicide mixtures and comparative sensitivity of tropical benthic microalgae. Mar. Pollut. Bull. 60, 1978–1987 (2010). 20800855 10.1016/j.marpolbul.2010.07.031

[b24] Escher B. I. *et al.* Toxic equivalent concentrations (TEQs) for baseline toxicity and specific modes of action as a tool to improve interpretation of ecotoxicity testing of environmental samples. J. Environ. Monit. 10, 612–621 (2008). 18449398 10.1039/b800949j

[b25] Cantin N. E., van Oppen M. J. H., Willis B. L., Mieog J. C. & Negri A. P. Juvenile corals can acquire more carbon from high-performance algal symbionts. Coral Reefs 28, 405–414 (2009).

[b26] Cantin N. E., Negri A. P. & Willis B. L. Photoinhibition from chronic herbicide exposure reduces reproductive output of reef-building corals. Mar. Ecol. Prog. Ser. 344, 81–93 (2007).

[b27] Chesworth J. C., Donkin M. E. & Brown M. T. The interactive effects of the antifouling herbicides Irgarol 1051 and Diuron on the seagrass *Zostera marina* (L.). Aquat. Toxicol. 66, 293–305 (2004). 15129771 10.1016/j.aquatox.2003.10.002

[b28] Jones R. J. & Kerswell A. P. Phytotoxicity of photosystem II (PSII) herbicides to coral. Mar. Ecol. Prog. Ser. 261, 149–159 (2003).

[b29] Negri A. P., Flores F., Röthig T. & Uthicke S. Herbicides increase the vulnerability of corals to rising sea surface temperature. Limnol. Oceanog. 56, 471–485 (2011).

[b30] Haworth P. & Steinback K. E. Interaction of herbicides and quinone with the Q(b)-Protein of the diuron-resistant *Chlamydomonas reinhardtii* mutant Dr2. Plant Physiol. 83, 1027–1031 (1987). 16665318 10.1104/pp.83.4.1027 PMC1056495

[b31] Davis A. M., Lewis S. E., Brodie J. E. & Benson A. The potential benefits of herbicide regulation: A cautionary note for the Great Barrier Reef catchment area. Sci. Total Environ. 490, 81–92 (2014). 24840283 10.1016/j.scitotenv.2014.04.005

[b32] Bainbridge Z. T., Brodie J. E., Faithful J. W., Sydes D. A. & Lewis S. E. Identifying the land-based sources of suspended sediments, nutrients and pesticides discharged to the Great Barrier Reef from the Tully–Murray Basin, Queensland, Australia. Mar. Freshwater Res. 60, 1081–1090 (2009).

[b33] Kennedy K. *et al.* Long term monitoring of photosystem II herbicides – Correlation with remotely sensed freshwater extent to monitor changes in the quality of water entering the Great Barrier Reef, Australia. Mar. Pollut. Bull. 65, 292–305 (2012). 22154275 10.1016/j.marpolbul.2011.10.029

[b34] Davis D. E., Pillai C. G. P. & Truelove B. Effects of prometryn, diuron, fluometuron, and MSMA on Chlorella and two fungi. Weed Sci. 24, 587–593 (1976).

[b35] Mitchell A., Reghenzani J., Faithful J., Furnas M. & Brodie J. Relationships between land use and nutrient concentrations in streams draining a ‘wet-tropics’ catchment in northern Australia. Mar. Freshwater Res. 60, 1097–1108 (2009).

[b36] Packett R., Dougall C., Rohde K. & Noble R. Agricultural lands are hot-spots for annual runoff polluting the southern Great Barrier Reef lagoon. Mar. Pollut. Bull. 58, 976–986 (2009). 19303607 10.1016/j.marpolbul.2009.02.017

[b37] McMahon K. *et al.* Herbicide contamination and the potential impact to seagrass meadows in Hervey Bay, Queensland, Australia. Mar. Pollut. Bull. 51, 325–334 (2005). 15757731 10.1016/j.marpolbul.2004.10.045

[b38] Shaw C. M., Lam P. K. & Mueller J. F. Photosystem II herbicide pollution in Hong Kong and its potential photosynthetic effects on corals. Mar. Pollut. Bull. 57, 473–478 (2008). 18486951 10.1016/j.marpolbul.2008.04.002

[b39] Bester K. Effects of pesticides on seagrass beds. Helgoland Mar. Res. 54, 95–98 (2000).

[b40] Gilliom R. J., Barbash J. E., Kolpin D. W. & Larson S. J. Peer reviewed: testing water quality for pesticide pollution. Environ. Sci. Tech. 33, 164A–169A (1999). 10.1021/es992770k 21662415

[b41] Lewis S. E. *et al.* Herbicides: A new threat to the Great Barrier Reef. Environ. Pollut. 157, 2470–2484 (2009). 19349104 10.1016/j.envpol.2009.03.006

[b42] Shaw M. *et al.* Monitoring pesticides in the Great Barrier Reef. Mar. Pollut. Bull. 60, 113–122 (2010). 19818971 10.1016/j.marpolbul.2009.08.026

[b43] Gatidou G., Stasinakis A. S. & Iatrou E. I. Assessing single and joint toxicity of three phenylurea herbicides using Lemna minor and Vibrio fischeri bioassays. Chemosphere 119, S69–S74 (2015). 24821233 10.1016/j.chemosphere.2014.04.030

[b44] Benitez F. J., Real F. J., Acero J. L. & Garcia C. Photochemical oxidation processes for the elimination of phenyl-urea herbicides in waters. J Hazard. Mater. 138, 278–287 (2006). 16839678 10.1016/j.jhazmat.2006.05.077

[b45] Faust M. *et al.* Predicting the joint algal toxicity of multi-component s-triazine mixtures at low-effect concentrations of individual toxicants. Aquat. Toxicol. 56, 13–32 (2001). 11690628 10.1016/s0166-445x(01)00187-4

[b46] Porsbring T., Backhaus T., Johansson P., Kuylenstierna M. & Blanck H. Mixture toxicity from photosystem II inhibitors on microalgal community succession is predictable by concentration addition. Environ. Toxicol. Chem. 29, 2806–2813 (2010). 20836067 10.1002/etc.346

[b47] Berenbaum M. C. The expected effect of a combination of agents: the general solution. J. Theor. Biol. 114, 413–431 (1985). 4021503 10.1016/s0022-5193(85)80176-4

[b48] Moss A., Brodie J. & Furnas M. Water quality guidelines for the Great Barrier Reef World Heritage Area: a basis for development and preliminary values. Mar. Pollut. Bull. 51, 76–88 (2005). 15757710 10.1016/j.marpolbul.2004.10.052

[b49] Ralph P. J. Herbicide toxicity of *Halophila ovalis* assessed by chlorophyll fluorescence. Aquat. Bot. 66, 141–152 (2000).

[b50] Fairchild J. F., Ruessler D. S. & Carlson A. R. Comparative sensitivity of five species of macrophytes and six species of algae to atrazine, metribuzin, alachlor, and metolachlor. Environ. Toxicol. Chem. 17, 1830–1834 (1998).

[b51] Jones R. J., Muller J., Haynes D. & Schreiber U. Effects of herbicides diuron and atrazine on corals of the Great Barrier Reef, Australia. Mar. Ecol. Prog. Ser. 251, 153–167 (2003).

[b52] Muller R. *et al.* Rapid exposure assessment of PSII herbicides in surface water using a novel chlorophyll a fluorescence imaging assay. Sci. Total Environ. 401, 51–59 (2008). 18501956 10.1016/j.scitotenv.2008.02.062

[b53] Tischer W. & Strotmann H. Relationship between inhibitor binding of chloroplasts and inhibition of photosynthetic electron transport. Biochim. Biophys. Acta 460, 113–125 (1977). 856261 10.1016/0005-2728(77)90157-8

[b54] Genty B., Briantais J.-M. & Baker N. R. The relationship between the quantum yield of photosynthetic electron transport and quenching of chlorophyll fluorescence. Biochim. Biophys. Acta 990, 87–92 (1989).

[b55] Knauert S., Escher B., Singer H., Hollender J. & Knauer K. Mixture toxicity of three photosystem II inhibitors (atrazine, isoproturon, and diuron) toward photosynthesis of freshwater phytoplankton studied in outdoor mesocosms. Environ. Sci. Tech. 42, 6424–6430 (2008). 10.1021/es072037q 18800510

[b56] de Zwart D. & Posthuma L. Complex mixture toxicity for single and multiple species: Proposed methodologies. Environ. Toxicol. Chem. 24, 2665–2676 (2005). 16268170 10.1897/04-639r.1

[b57] Bengtson Nash S. M., McMahon K., Eaglesham G. & Müller J. F. Application of a novel phytotoxicity assay for the detection of herbicides in Hervey Bay and the Great Sandy Straits. Mar. Pollut. Bull. 51, 351–360 (2005). 15757734 10.1016/j.marpolbul.2004.10.017

[b58] Shaw C. M., Brodie J. & Mueller J. F. Phytotoxicity induced in isolated zooxanthellae by herbicides extracted from Great Barrier Reef flood waters. Mar. Pollut. Bull. 65, 355–362 (2012). 22370098 10.1016/j.marpolbul.2012.01.037

[b59] Magnusson M., Heimann K., Ridd M. & Negri A. P. Pesticide contamination and phytotoxicity of sediment interstitial water to tropical benthic microalgae. Water Res. 47, 5211–5221 (2013). 23870432 10.1016/j.watres.2013.06.003

[b60] Anthony K. R. N. & Kerswell A. P. Coral mortality following extreme low tides and high solar radiation. Mar. Biol. 151, 1623–1631 (2007).

[b61] Bandow N., Altenburger R. & Brack W. Application of nd-SPME to determine freely dissolved concentrations in the presence of green algae and algae-water partition coefficients. Chemosphere 79, 1070–1076 (2010). 20385402 10.1016/j.chemosphere.2010.03.021

[b62] OECD. Guidance document on aquatic toxicity testing of difficult substances and mixtures. OECD Series on Testing and Assessment. No. 23. (2000) Available at http://www.oecd.org/officialdocuments/publicdisplaydocumentpdf/?doclanguage=en&cote=env/jm/mono%282000%296 (Accessed: 23rd October 2015).

[b63] Schreiber U. Pulse-Amplitude-Modulation (PAM) Fluorometry and Saturation Pulse Method: An Overview in Chlorophyll a Fluorescence Vol. 19 Advances In Photosynthesis and Respiration (ed Papageorgiou G. C. ) Ch. 11, 279–319 (Springer: Netherlands, , 2004).

[b64] Tomlin C. The pesticide manual: A world compendium (12th edition). British Crop Protection Council, Farnham, Surrey, UK, 1250 p (2000).

[b65] Haynes D., Ralph P., Prange J. & Dennison B. The Impact of the Herbicide Diuron on Photosynthesis in Three Species of Tropical Seagrass. Mar. Pollut. Bull. 41, 288–293 (2000).

[b66] van Dam J. W., Uthicke S., Beltran V. H., Mueller J. F. & Negri A. P. Combined thermal and herbicide stress in functionally diverse coral symbionts. Environ. Pollut. 204, 271–279 (2015). 25989453 10.1016/j.envpol.2015.05.013

[b67] Sikka H. & Pramer D. Physiological effects of fluometuron on some unicellular algae. Weed Sci. 16, 296–299 (1968).

[b68] Jones R. J. & Kerswell A. P. Phytotoxicity of Photosystem II (PSII) herbicides to coral. Marine Ecology Progress Series 261, 149–159 (2003).

[b69] Brock T. C. M. *et al.* Comparing aquatic risk assessment methods for the photosynthesis-inhibiting herbicides metribuzin and metamitron. Environ. Pollut. 130, 403–426 (2004). 15182972 10.1016/j.envpol.2003.12.022

[b70] Seery C. R., Gunthorpe L. & Ralph P. J. Herbicide impact on *Hormosira banksii* gametes measured by fluorescence and germination bioassays. Environ. Pollut. 140, 43–51 (2006). 16143437 10.1016/j.envpol.2005.07.001

[b71] ANZECC & ARMCANZ. *Australian and New Zealand guidelines for fresh and marine water quality.* Australian and New Zealand Environment and Conservation Council and Agriculture and Resource Management Council of Australia and New Zealand. (2000) Availabe at http://www.environment.gov.au/water/publications/quality/nwqms-guidelines-4-vol1.html. (Accessed 22 October 2015).

[b72] Smith R. A. *et al.* Proposed guideline values for six priority pesticides of the Great Barrier Reef and its adjacent catchments. 40 p (Department of Science, Information Technology, Innovation and the Arts, Queensland Government, Brisbane, 2015).

